# Combined Endoscopic and Laparoscopic Management of Postcholecystectomy Mirizzi Syndrome from a Remnant Cystic Duct Stone: Case Report and Review of the Literature

**DOI:** 10.1155/2016/1896368

**Published:** 2016-03-07

**Authors:** Arpit Amin, Yuriy Zhurov, George Ibrahim, Anthony Maffei, Jonathan Giannone, Thomas Cerabona, Ashutosh Kaul

**Affiliations:** Department of Surgery, New York Medical College-Westchester Medical Center, Valhalla, NY 10595, USA

## Abstract

Mirizzi syndrome has been defined in the literature as common bile duct obstruction resulting from calculi within Hartmann's pouch or cystic duct. We present a case of a 78-year-old female, who developed postcholecystectomy Mirizzi syndrome from a remnant cystic duct stone. Diagnosis of postcholecystectomy Mirizzi syndrome was made on endoscopic retrograde cholangiography (ERCP) performed postoperatively. The patient was treated with a novel strategy by combining advanced endoscopic and laparoscopic techniques in three stages as follows: Stage 1 (initial presentation): endoscopic sphincterotomy with common bile duct stent placement; Stage 2 (6 weeks after Stage 1): laparoscopic ultrasonography to locate the remnant cystic duct calculi followed by laparoscopic retrieval of the calculi and intracorporeal closure of cystic duct stump; Stage 3 (6 weeks after Stage 2): endoscopic removal of common bile duct stent along with performance of completion endoscopic retrograde cholangiogram. In addition, we have performed an extensive review of the various endoscopic and laparoscopic management techniques described in the literature for the treatment of postcholecystectomy syndrome occurring from retained cystic duct stones.

## 1. Introduction

Mirizzi syndrome has been defined as bile duct obstruction from stone impaction in Hartman's pouch or cystic duct [1]. Mirizzi syndrome has been well-described in the literature in patients with a reported incidence of 0.7–1.4% [2]. However, there is a paucity of literature describing Mirizzi syndrome after patients have undergone cholecystectomy and subsequent management of this entity.

We describe a minimally invasive combined endoscopic and laparoscopic management of Mirizzi syndrome occurring from a retained cystic duct stump stone in a patient after laparoscopic cholecystectomy. In addition, a literature review on the topic of retained cystic duct stone after cholecystectomy was performed to highlight the diagnostic and therapeutic options available in management of this entity.

## 2. Case Presentation

A 78-year-old female was referred to the surgical service for elective cholecystectomy after suffering from a 2nd episode of right upper quadrant abdominal pain due to chronic cholecystitis within three months. The patient's past medical history was significant for hypertension and gastroesophageal reflux. Her vital signs were normal. Physical exam revealed minimal right upper quadrant tenderness with palpation and no jaundice. Her preoperative lab profile, including white blood cell count and liver function tests, was normal (Table 1). Preoperative ultrasound revealed gallstones, normal gallbladder wall thickness, and a normal common bile duct diameter.

During laparoscopic cholecystectomy, dissection of the cystohepatic triangle was noted to be challenging due to inflammation. An intraoperative cholangiogram was attempted; however some resistance was noted when the cholangiogram catheter was being fed into the cystic duct. At this point, the cholangiogram was aborted and the cystic duct was closed with 2-0 Vicryl suture in a running fashion. Subtotal cholecystectomy was performed. A Jackson Pratt (JP) drain was placed in the gallbladder fossa. The patient was discharged home on postoperative day one with a JP drain in place after tolerating a fat-restricted diet. The JP drain was removed during outpatient follow-up visit a week after the surgery due to minimal output.

**Figure 1:**
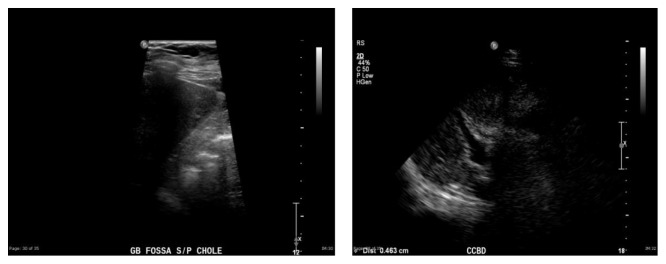
Postoperative abdominal ultrasound showing no evidence of collection and distal common bile duct diameter of 4.6 mm.

**Figure 2:**
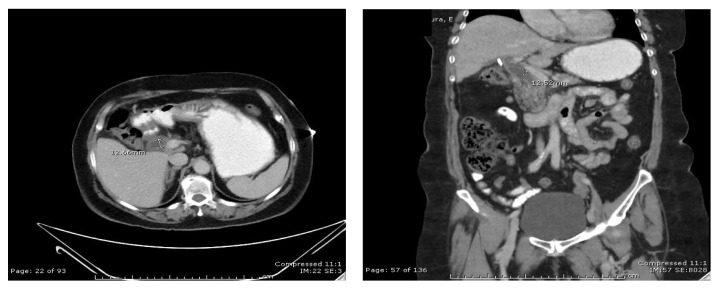
Postoperative CT scan abdomen with oral and IV contrast (axial and coronal views) showing dilated common hepatic duct (12.5 mm diameter).

**Figure 3:**
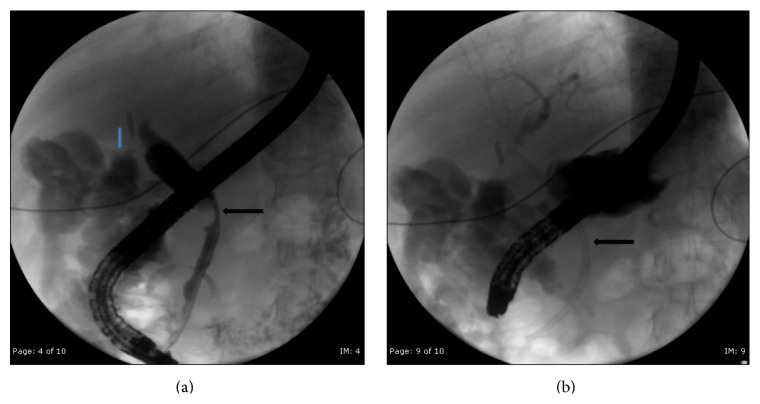
(a) ERCP showing compression of common hepatic duct from remnant cystic duct stone (black arrow) and bile leak in background (blue arrow). (b) Fluoroscopic image after stent placement (black arrow).

Five days after removal of the JP drain, the patient returned to the emergency department with right upper quadrant abdominal pain, nausea, vomiting, and intermittent fevers. Laboratory studies revealed elevation of liver function tests with normal lipase (Table 1). Abdominal ultrasound did not reveal any gallbladder fossa collection (Figure 1). Computerized tomography of the abdomen and pelvis revealed central intrahepatic biliary duct dilation and the common hepatic duct was dilated measuring up to 1.2 cm (Figure 2). Based on this presentation, the decision was made to perform an endoscopic retrograde cholangiopancreatography (ERCP). The ERCP revealed a single large 2 cm stone in the cystic duct remnant causing stenosis of the common bile duct consistent with Mirizzi syndrome along with extravasation of contrast from cystic duct stump (Figure 3). Sphincterotomy and common bile duct stent placement was performed to temporarily relieve the obstructive pathology and biliary leak. The patient's symptoms and liver function tests showed improvement after the placement of common bile duct stent (Table 1). The patient was discharged home on a fat-restricted diet and brought to the operating room for laparoscopic treatment of Mirizzi syndrome from retained cystic duct stone 6 weeks after the initial operation.

**Figure 4:**
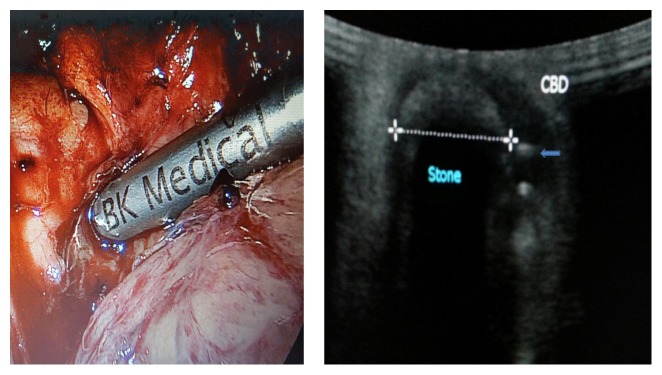
Laparoscopic ultrasonography probe utilized to determine location of remnant cystic duct stone and common bile duct stent (blue arrow).

**Figure 5:**
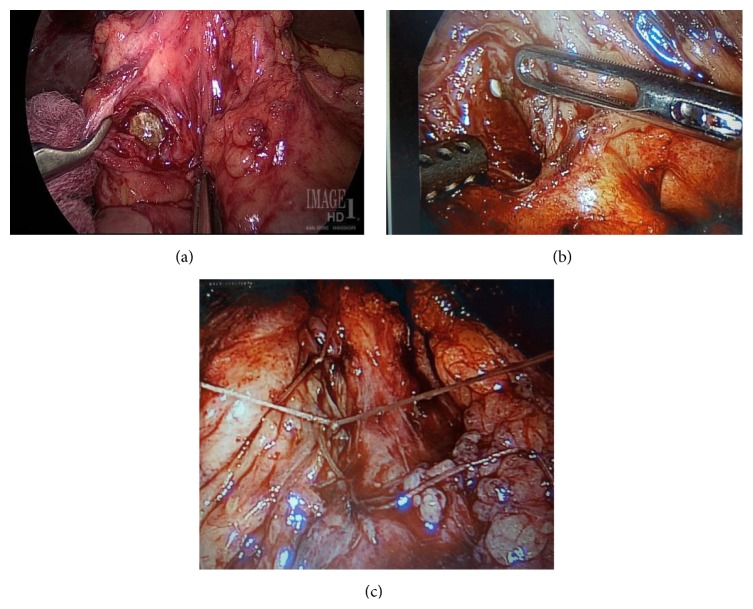
(a) Laparoscopic retrieval of remnant cystic duct stone. (b) Plastic common bile duct stent (pointed out by grasper) visible through the junction of cystic duct and common bile duct. (c) Laparoscopic intracorporeal closure of remnant cystic duct stump with absorbable suture.

**Figure 6:**
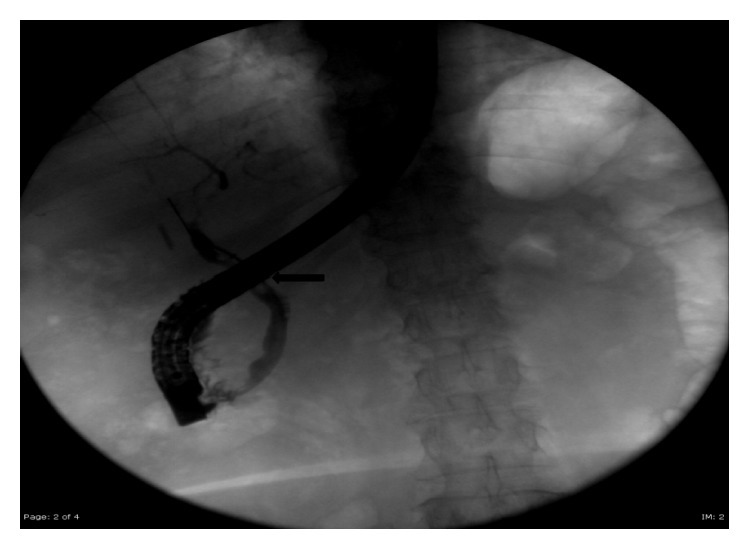
Post-op ERCP demonstrating resolution of Mirizzi syndrome and no cystic duct stump leak. Balloon sweep (black arrow) of common bile duct was performed after stent removal and it revealed no common bile duct stones.

During the 2nd operation, we utilized intraoperative laparoscopic ultrasonography to clearly define the presence of cystic duct remnant stone and the common bile duct stent to guide the laparoscopic dissection (Figure 4). An incision was made directly over the cystic duct remnant and the cystic duct stone was removed (Figure 5). The cystic duct remnant was then closed with 2-0 Vicryl sutures via intracorporeal suturing technique. A drain was placed in the gallbladder fossa. The patient was placed on fat-restricted diet postoperatively. No bile leak was detected from the drain and the drain was removed prior to discharge. The patient returned to the hospital electively six weeks after discharge for repeat ERCP. On repeat ERCP, the common bile duct stent was removed. Post-stent-removal cholangiogram revealed normal biliary tract appearance with resolution of previously noted Mirizzi syndrome (Figure 6). On outpatient follow-up, the patient is asymptomatic and has normal liver function tests after removal of the stent (Table 1).

## 3. Discussion

An estimated 750,000 cholecystectomies are performed in the United States every year [3]. Approximately 10–30% of patients continue to suffer from the same constellation of symptoms that were present before their operation. This group of patients is categorized as suffering from postcholecystectomy syndrome [14]. Patients with postcholecystectomy syndrome may present with wide spectrum of persistent signs and symptoms such as abdominal pain, fever, jaundice, pruritus, nausea, and vomiting after cholecystectomy [3, 4, 9, 10]. It is important to consider the possibility of biliary as well as extrabiliary etiologies while evaluating patients with postcholecystectomy syndrome. Extrabiliary sources of postcholecystectomy syndrome include pneumonia, acute coronary syndrome, hepatocellular disease, pancreatic disease, gastroesophageal reflux, and peptic ulcer disease [4]. Similarly, biliary sources of postcholecystectomy syndrome like biliary injury, biliary stricture, retained stone within the common bile duct, sphincter of Oddi stenosis, retained stone within remnant gallbladder, and retained stone within cystic duct remnant must be entertained [4]. Our case reports highlight the fact that Mirrizzi syndrome from retained cystic duct stump stone should, also, be considered as part of the differential diagnosis of postcholecystectomy syndrome.

The role of cystic duct remnant length as a cause of postcholecystectomy syndrome has been debated and studied within the literature. Cystic duct stump remnant has been defined as residual cystic duct stump length greater than 1 cm in the literature [16]. Bodvall and Overgaard have reported that a cystic duct remnant larger than 1 cm was present in 67% of patients with common bile duct stones after cholecystectomy [17]. Rogy et al. reported that the role of cystic duct stump remnant as a reason for reoperation after cholecystectomy is negligible [18]. While a long cystic duct stump remnant by itself may not be related to postcholecystectomy syndrome, presence of any remnant stones within the cystic duct stump may lead to postcholecystectomy syndrome. This is an important issue to consider in the laparoscopic era wherein the tendency to leave a long cystic duct stump and/or perform subtotal cholecystectomy is increasingly favored to avoid injury to the common bile duct. Palanivelu et al. have reported an incidence of remnant cystic duct calculi of 4.19% in patients undergoing laparoscopic cholecystectomy as compared to an incidence of 0.02% in patients undergoing conventional open cholecystectomy [9].

Diagnosis of remnant cystic duct stone as the source of postcholecystectomy syndrome requires high index of suspicion. Patients may either present immediately within the first week after the operation as reported in our case report or present as late as after 32 years as reported in the literature [13]. Laboratory testing may demonstrate leukocytosis along with derangements in liver function tests (total and direct bilirubin, alkaline phosphatase, gamma-glutamyl transferase, aspartate aminotransferase, and alanine aminotransferase) [3]. Phillips et al. demonstrated that 75% of these patients had derangement of at least one standard liver function test parameter and noted that gamma-glutamyl transferase was the most common abnormality observed in 80% of patients with remnant cystic duct stone [3].

Given the broad differential diagnosis for the cause of postcholecystectomy syndrome, a combination of noninvasive radiologic imaging in the form of abdominal ultrasonography, abdominal CT scan with IV contrast, and magnetic resonance cholangiopancreatography (MRCP) should be considered to identify remnant cystic duct calculus as the cause of postcholecystectomy syndrome. Abdominal ultrasonography will demonstrate intrahepatic and extrahepatic biliary ductal dilation proximal to remnant cystic duct calculus along with normal caliber biliary duct distal to the site of obstruction [8]. In the case series reported by Palanivelu et al., abdominal ultrasonography was able to identify remnant cystic duct calculus in 9 out of 15 patients (sensitivity 60%) [9]. Similarly, in the case series by Phillips et al., remnant cystic duct calculus was identified with abdominal ultrasonography in 5 out of 9 patients (sensitivity 55%) [3]. MRCP will demonstrate the characteristic finding of remnant cystic duct calculus compressing the common hepatic duct with proximal ductal dilation and normal size distal common bile duct [8]. In the case series reported by Palanivelu et al., MRCP was able to identify remnant cystic duct calculus in all 15 patients (sensitivity 100%) [9]. On the other hand, in the case series by Phillips et al., remnant cystic duct calculus was identified in 8 out of 9 patients (sensitivity 89%) [3]. Interestingly, in the case series reported by Walsh et al., MRCP was utilized only in 1 out of 7 patients and it failed to demonstrate remnant cystic duct calculus [4]. Abdominal computed tomography (CT) may, also, be considered as an alternative in institutions where MRCP is not available. Abdominal CT may reveal a smooth tapering of the common bile duct in the absence of pancreatic mass along with identifying any remnant cystic duct calculus or gallbladder bed collections [8]. In addition, abdominal CT with IV contrast may identify any vascular injury to hepatic pedicle. In the case series by Phillips et al., 7 patients underwent abdominal CT and a remnant cystic duct calculus was identified in 5 patients (sensitivity 71%) [3]. In conclusion, MRCP appears to be the most sensitive noninvasive imaging modality available currently to identify remnant cystic duct calculi in patients suffering from postcholecystectomy syndromes. However, abdominal ultrasonography can still be employed as the initial noninvasive modality to identify remnant cystic duct calculi in patients presenting with postcholecystectomy syndrome since it is more readily available and less expensive than MRCP.

Management options for postcholecystectomy Mirizzi syndrome are largely determined based on underlying problem (i.e., stone within gallbladder remnant or stone within remnant cystic duct stump). It is imperative to attempt to determine the etiology of postcholecystectomy Mirizzi syndrome based on review of initial operative reports and preoperative imaging studies. Special attention should be paid to operative details like hostile operative conditions due to acute inflammation, performance of intraoperative cholangiogram, milking of cystic duct towards gallbladder before ligation, presence of dilated or long cystic duct, utilization of special techniques like stapling or ligation loops for cystic duct ligation, and performance of subtotal cholecystectomy [3, 9]. Close attention to these intraoperative details may help differentiate if postcholecystectomy Mirizzi syndrome is due to a stone within gallbladder remnant or due to a stone within remnant cystic duct.

If the postcholecystectomy Mirizzi syndrome is due to stone within gallbladder remnant after subtotal cholecystectomy, surgical treatment with completion cholecystectomy and retrieval of gallbladder calculi with either laparoscopic or open techniques should be offered [4]. Endoscopic retrieval of retained gallbladder remnant calculi should not be entertained [4].

On the other hand, if there is high index of suspicion that postcholecystectomy Mirizzi syndrome is due to stone within remnant cystic duct stump, endoscopic retrograde cholangiography (ERCP) techniques may be employed initially [3]. Examples of ERCP techniques that have been described in the literature for remnant cystic duct stone retrieval include (i) sphincterotomy combined with traditional balloon and basket use for stone retrieval, (ii) sphincterotomy combined with application of extracorporeal shock wave lithotripsy (ESWL) followed by endoscopic retrieval of fragments, and (iii) sphincterotomy combined with endoscopic transpapillary application of holmium laser for stone fragmentation followed by endoscopic retrieval of fragments (Table 2) [3–7]. Technical factors like size of the remnant cystic duct, location of stone within the cystic duct, and presence of acute inflammation may contribute to difficulty with stone extraction with traditional balloon or basket use [4]. In difficult cases, stepwise endoscopic treatment starting with sphincterotomy and/or endobiliary stenting followed by remnant cystic duct stone fragmentation with ESWL or laser application and subsequent endoscopic removal of fragmented calculi may be employed [5]. However, it must be mentioned that availability of endoscopic treatment of postcholecystectomy Mirizzi syndrome from remnant cystic duct calculi may be institution specific due to high level of endoscopic expertise required to execute this management strategy. Historically, open surgical treatment has been offered for remnant cystic duct stump stones (Table 3) [3, 4, 12]. However, with the advent of minimally invasive techniques, laparoscopic retrieval of remnant cystic duct stump calculi and intracorporeal laparoscopic suturing has been shown to be effective treatment for remnant cystic duct stump calculi causing postcholecystectomy syndrome (Table 3) [9].

In our case report, we have described a hybrid endoscopic and laparoscopic approach to the management of postcholecystectomy Mirizzi syndrome from remnant cystic duct calculi. Based on our review of the published literature of endoscopic and surgical management of postcholecystectomy syndrome from remnant cystic duct calculi, this approach has not been described before. In our case, we opted to first perform endoscopic sphincterotomy with endobiliary stent placement to temporarily relieve the patient's symptoms from Mirizzi syndrome while allowing for acute inflammation and biliary leak within the postcholecystectomy operative bed to resolve. Secondly, we opted to utilize laparoscopic intraoperative ultrasonography to localize the endobiliary stent and remnant cystic duct calculi. The combination of direct laparoscopic visualization and intraoperative ultrasonography ensured that our dissection stayed on top of the remnant cystic duct stump and away from the common bile duct. We believe that intraoperative laparoscopic ultrasonography should be utilized liberally in these cases of postcholecystectomy Mirizzi syndrome to avoid any injury to common bile duct. Lastly, intracorporeal closure of the remnant cystic duct stump was performed after stone retrieval leaving the endobiliary prosthesis in place for additional six weeks. We believe that preoperative endobiliary prosthesis can help avoid common bile duct exploration in a hostile operative field along with resolving any cystic stump leak that may occur after intracorporeal closure. Subsequently, endobiliary prosthesis can be removed postoperatively once adequate time (i.e., six weeks) has elapsed after intracorporeal closure of the cystic duct stump. The advantage of this combined endoscopic and laparoscopic approach is that it allows for the acute inflammatory stage to resolve thereby allowing for minimally invasive treatment of a complex problem while avoiding common bile duct exploration. In addition, this approach may also be employed at institutions where endoscopic expertise regarding extracorporeal shock wave lithotripsy and laser fragmentation techniques may not be available. The disadvantage of this hybrid approach is that the patient will likely have to undergo three procedures (preoperative ERCP with sphincterotomy with stent, laparoscopic stone retrieval, postoperative ERCP, and stent removal) when compared to single-stage laparoscopic approach recommended by Palanivelu et al. in their case series.

Prevention of postcholecystectomy Mirizzi syndrome from remnant cystic duct stump calculi requires meticulous attention to operative technique during index laparoscopic cholecystectomy. Close attention to correct identification of cystic duct-gallbladder junction, milking of the cystic duct back to gallbladder prior to ligation, consideration for performance of selective intraoperative cholangiography in cases with long or dilated cystic duct, and intraoperative removal of any detected cystic duct stump calculi during index operation may help prevent occurrence of remnant cystic duct stump calculi [3, 4, 9]. However, if the operative field is hostile with obliteration of cystohepatic triangle, a subtotal laparoscopic cholecystectomy or conversion to open cholecystectomy in combination with intraoperative cholangiography may be a safer option since postcholecystectomy Mirizzi syndrome from remnant cystic duct calculi can be managed endoscopically in these cases while avoiding risk of common bile duct injury [3, 4, 9].

## 4. Conclusion

Mirizzi syndrome due to remnant cystic duct calculi should be considered in the differential diagnosis of patients presenting with postcholecystectomy syndrome. Diagnosis of this rare entity can be confirmed with the help of abdominal ultrasonography and MRCP in the correct clinical scenario. ERCP with sphincterotomy and stenting in combination with ESWL or laser lithotripsy may be offered as first-line therapy if endoscopic expertise is available for this complex problem. While laparoscopic removal of remnant cystic duct calculi with common bile duct exploration and closure has been described in the literature, this approach as first-line therapy may only be possible at high-volume centers with advanced laparoscopic expertise. In this case report, we have described hybrid stepwise endoscopic and laparoscopic management in combination with use of intraoperative laparoscopic ultrasonography highlighting a novel approach to the management of postcholecystectomy Mirizzi syndrome from remnant cystic duct calculi.

## Figures and Tables

**Table 1:** Laboratory profile during patient course.

	WBC (K/CU mm) (4.8–10.8)	T. bil. (mg/dL) (0.2–1.3)	D. bil. (mg/dL) (0.1–0.6)	AST (U/L) (4–35)	ALT (U/L) (6–55)	ALP (U/L) (40–150)	GGT (U/L) (9–36)
Index presentation	5.6	0.3	Not measured	22	15	101	31
Two weeks after laparoscopic cholecystectomy	6.3	2.1	1.5	968	723	329	Not measured
One week after ERCP and stent placement	4.7	1.2	0.7	115	274	281	Not measured
Prior to laparoscopic removal of remnant cystic duct stone	9.9	0.6	0.3	32	26	81	Not measured
After ERCP and stent removal	5.1	0.6	Not measured	20	17	89	Not measured

**Table 2:** Review of literature describing endoscopic management of postcholecystectomy syndrome from remnant cystic duct calculi.

	Number of patients with postcholecystectomy from remnant cystic duct calculi	Mean time of presentation from initial operation	Type of initial operation and indication	Indication for initial operation	Endoscopic management technique	Outcome at follow-up
Phillips et al. [3]	12 (5 out of 12 patients underwent endoscopic management)	34.2 months	Laparoscopic cholecystectomy in 9 patients Open cholecystectomy in 3 patients	Acute cholecystitis in 5 patients Chronic cholecystitis in 7 patients	(i) ERCP with sphincterotomy and balloon extraction in 3 patients (ii) ERCP retrieval of stones after extracorporeal shock lithotripsy in 2 patients (iii) 1 patient refused surgical intervention after failed ERCP	Follow-up available in 9 patients Resolution of symptoms in 7 patients out of these 9 patients at mean follow-up of 11.8 months

Walsh et al. [4]	7 (2 out of 7 patients underwent endoscopic management)	8.5 years	Laparoscopic cholecystectomy in 4 patients Open cholecystectomy in 3 patients	Acute cholecystitis in 2 patients Chronic cholecystitis in 5 patients	(i) ERCP retrieval of stones after extracorporeal shock wave lithotripsy in 1 patient (ii) ERCP retrieval of stones after holmium laser fragmentation in 1 patient	All patients were symptom-free at mean follow-up of 31 months

Benninger et al. [5]	3 (3 out of 3 patients underwent endoscopic management)	2.5 months	Laparoscopic cholecystectomy in 2 patients Open cholecystectomy in 1 patient	Acute cholecystitis in 2 patients Chronic cholecystitis with choledocholithiasis in 1 patient	(i) ERCP retrieval of stones after extracorporeal shock wave lithotripsy in 3 patients	All patients were symptom-free at mean follow-up of 62 months

Kodali and Petersen [6]	2 (2 out of 2 patients underwent endoscopic management)	2 years	Laparoscopic cholecystectomy in 2 patients	Chronic cholecystitis in 2 patients	ERCP retrieval of stone with balloon and basket retrieval technique	All patients were symptom-free at discharge

Janes et al. [7]	1	1 year	Laparoscopic cholecystectomy	Chronic cholecystitis	Stone migration into common bile duct during treatment planning with subsequent ERCP retrieval	Symptom-free at 3-month follow-up

Wani et al. [8]	1	3 years	Laparoscopic cholecystectomy	Chronic cholecystitis	ERCP mechanical lithotripsy of remnant cystic duct stone and retrieval of stone fragments with balloon	Symptom-free with resolution of LFTs at discharge

**Table 3:** Review of literature describing surgical management of postcholecystectomy syndrome from remnant cystic duct calculi.

	Number of patients with postcholecystectomy Mirizzi syndrome	Mean time of presentation from initial operation	Type of initial operation and indication	Indication for initial operation	Surgical management of postcholecystectomy Mirizzi syndrome	Outcome at follow-up
Palanivelu et al. [9]	15 (all 15 patients underwent surgical management)	8 months	Laparoscopic subtotal cholecystectomy in 13 patients Conventional laparoscopic cholecystectomy in 2 patients	Acute cholecystitis in 13 patients Indication not mentioned in 2 patients undergoing conventional cholecystectomy	*Laparoscopic* retrieval of remnant cystic duct stone in all patients (i) Primary closure of common bile duct in 11 patients (ii) Closure of common bile duct around T-tube in 4 patients	CBD stone in 1 patient post-op managed with ERCP and stent Biliary pancreatitis in 1 patient All patients were symptom-free at 3-month follow-up

Phillips et al. [3]	12 (6 out of 12 patients underwent surgical management)	34.2 months	Laparoscopic cholecystectomy in 9 patients Open cholecystectomy in 3 patients	Acute cholecystitis in 5 patients Chronic cholecystitis in 7 patients	(i) *Open* retrieval of cystic duct remnant calculi and *open* common bile duct exploration in 6 patients (ii) 1 patient refused surgical intervention after failed ERCP	Follow-up available in 9 patients Resolution of symptoms in 7 patients out of these 9 patients at mean follow-up of 11.8 months

Walsh et al. [4]	7 (5 out of 7 patients underwent surgical management)	8.5 years	Laparoscopic cholecystectomy in 4 patients Open cholecystectomy in 3 patients	Acute cholecystitis in 2 patients Chronic cholecystitis in 5 patients	(i) *Open* retrieval of cystic duct remnant stone in 4 patients (ii) *Laparoscopic* retrieval of cystic duct remnant stone in 1 patient	All patients were symptom-free at mean follow-up of 31 months

Tantia et al. [10]	7 (7 out of 7 patients underwent surgical management)	12.8 years	Open cholecystectomy in 6 patients Laparoscopic cholecystectomy in 1 patient	Not known	*Laparoscopic* completion cholecystectomy in 7 patients with laparoscopic common bile duct exploration in 2 patients (primary CBD closure in 1 patient and choledochoduodenostomy in 1 patient)	All patients were symptom-free at 3-month follow-up

Chowbey et al. [11]	3 (3 out of 3 patients underwent surgical management)	7.6 months	Laparoscopic subtotal cholecystectomy in 3 patients	Not known	*Laparoscopic* completion cholecystectomy in 3 patients	All patients were symptom-free at mean follow-up of 2.3 years

Endo et al. [12]	1	8 years	Open cholecystectomy	Not known	*Open* retrieval of cystic duct remnant stone and closure of CBD defect with T-tube placement	Symptom-free with resolution of common bile duct stricture at 9-month follow-up

Gurel et al. [13]	1	32 years	Open cholecystectomy	Acute cholecystitis	*Laparoscopic* completion cholecystectomy and retrieval of gallbladder remnant stone	Symptom-free at discharge with resolution of LFTs at discharge

Pernice and Andreoli [14]	1	16 years	Laparoscopic cholecystectomy	Chronic cholecystitis	*Laparoscopic* completion cholecystectomy of gallbladder remnant	Symptom-free at 8-month follow-up

Sahoo and Kumar [15]	1	6 years	Laparoscopic cholecystectomy	Not known	*Laparoscopic* retrieval of cystic duct stump stone and intracorporeal closure of stump	Symptom-free at discharge
